# Impact of the COVID-19 epidemic on medical product imports from china from outbreak to stabilization: Monthly panel data regression and instrumental variable test

**DOI:** 10.3389/fpubh.2023.1115650

**Published:** 2023-02-09

**Authors:** Yuanjie Pu, Aidi Xu, Hang Wang, Fangbin Qian

**Affiliations:** ^1^School of Hospitality Administration, Zhejiang Yuexiu University, Shaoxing, China; ^2^Center for International Education, Philippine Christian University, Manila, Philippines

**Keywords:** COVID-19 epidemic, trade in medical products, trade diversion, global public health governance, political relation

## Abstract

This study conduct regressions of panal data with OLS and test with IV, empirically examines the COVID-19 epidemic's impact on the import of medical products from China from the perspective of the importing countries, exporting country, and other trading partners, and analyzes the inter-temporal impact across different product categories. The empirical results reveal that, in importing countries, the COVID-19 epidemic increased the import of medical products from China. In China, as an exporting country, the epidemic inhibited the export of medical products; by contrast, for other trading partners, it promoted the import of medical products from China. Among them, key medical products were most affected by the epidemic, followed by general medical products and medical equipment. However, the effect was generally found to wane after the outbreak period. Additionally, we focus on how political relations shape China's medical product export pattern and how the Chinese government is using trade means to improve external relations. In the post-COVID-19 era, countries should prioritize the stability of supply chains for key medical products and actively engage in international cooperation on health governance to further combat the epidemic.

## 1. Introduction

Coronavirus disease 2019 (COVID-19) began spreading in late 2019 and quickly spread globally, with large-scale outbreaks beginning in numerous regions worldwide in March 2020 and continuing to this day. According to the World Health Organization COVID-19 Dashboard, by October 2022, more than 600 million COVID-19 cases had been confirmed globally and nearly 7 million people had died from the virus—a catastrophe for mankind. Moreover, the pandemic has significantly impacted the global commodity trade and has hindered the trade liberalization process (1–4). According to World Trade Organization statistics, the volume of the global commodity trade decreased by 7.3% year-on-year in 2020. Despite the negative growth in overall trade, the global trade volume of medical products buck the trend, with an average growth rate of over 14% in 2020 and 2021, and its share in total trade in goods increased from 5.3% before the pandemic to 6.6% in 2020 and 5.9% in 2021.

This study empirically examines the epidemic's impact on the import of medical products from China from January 2020 to September 2022. There are two main reasons for selecting China. First, China is the largest commodity exporter in the world and has the reputation of a “global factory.” It also has trade relations with almost all countries worldwide; therefore, the analysis of imports from China exhibits a certain universality. Second, when the global epidemic broke out in March 2020, China's production capacity gradually recovered. According to a report by The State Council of China, by April 2020, the daily output of personal protective equipment had increased to 90 times the level in January 2020,^1^ which compensated the trade restriction measures imposed by China in the early stage of the global pandemic.^2^

This study considers the following three types of medical products: The first are critical medical products directly used in the prevention, control, and treatment of COVID-19, including medical masks, protective clothing, disinfectants, and nucleic acid test kits, which were largely in short supply in the early stages of the outbreak. For epidemic prevention and control, governments of all countries have greatly expanded the public health procurement of key medical products, especially personal protective goods (5). From the public's perspective, the spread of the pandemic has caused a sharp rise in the risk of global uncertainty (6). The resulting social panic caused residents to bulk-buy masks and other personal protective products, thereby increasing the import demand for such products. The second category of products are pharmaceutical products—namely, all products covered by Chapter 30 of the Harmonized System of Customs Codes. The spread of COVID-19 has seriously affected residents' health. The treatment of COVID-19 and complications and sequelae of the pneumonia require significant drug support; hence, the demand for drugs in various countries has also increased greatly. The third type of products are medical equipment (including ventilators)—that is, the tools used by public health institutions to diagnose and treat diseases. Such products are characterized by high technology intensity and are predominantly exported from developed countries in Europe and from the United States, with fewer exports from China.

The pandemic has a profound impact on the trade flow and trade pattern of the world's medical products. At the same time, as a means of allocating medical resources among countries, the trade of medical products is an important guarantee for international cooperation to fight the epidemic. Therefore, the research in this field has strong academic value and practical significance.Research in this field will help provide practical reference for international health governance cooperation In the field of medical product trade in the context of the pandemic, the existing research predominantly focuses on the following four aspects: The first is studying the direct impact of the pandemic on the pattern of imports and exports of medical products. For instance, Soyyigit and Eren (7) and Mehrotra et al. (8) focus on the problems in the supply chain of medical products under the impact of COVID-19, finding that the current global value chain structure of medical products is not immune to the impact of supply shocks in emergency situations, such as the COVID-19 pandemic. The second aspect concerns the impact of medical trade policies on the background of the pandemic. Owing to the soaring demand for medical products, numerous countries have adopted measures to encourage imports and restrict exports in the early stages of the outbreak. However, the effectiveness of these policies is questionable, because they are not conducive to the healthy development of the industry in the long run (9, 10). The third aspect examines the role of political relations in the allocation of medical products among countries. The scarcity of medical products makes their international trade have a stronger political meaning (11). Further, desirable political relations were proven conducive to the import and export of medical products between countries during the early stages of the pandemic (12). The fourth aspect is the focus on pandemic control and prevention strategies in the post-pandemic era; era. Shang et al. (13) first recognize the positive role of the involvement of government authorities in mitigating the impact of the epidemic. Yin et al. (14) believe that ensuring the stability of the supply chain of important products is the basis of the fight against the epidemic. The closest studies to ours are those by Liu et al. (15) and Hayakawa and Mukunoki (1). The former focuses on the negative impact of the epidemic on imported goods from China; however, for medical products, this negative effect is offset by the demand effect. On this basis, this study draws the conclusion that, in importing countries, the epidemic promoted the import of medical products from China. The latter study discusses empirically the impact of the epidemic on the imports and exports of medical products worldwide, and expands the existing research under the following four dimensions: political, economic, population, and geographical connections. Additionally, we consider political factors but draw different conclusions.

Compared with the literature, the marginal contributions of this paper are as follows: First, previous studies on the impact of the epidemic on the imports and exports of medical products have been limited to the first few months or the first year of the outbreak. This study investigates, for the first time, the impact of the epidemic on the imports and exports of medical products over the entire period and studies the change in the impact among different periods, thereby filling a gap in terms of the period analyzed. Second, existing studies have neglected the two-way impact of the epidemic on medical product trade. This study uses the instrumental variable method to solve this problem, thus making the empirical evidence more rigorous and credible. Third, this study also focuses on the relationship between political relations and trade of medical products, thereby improving the integration of political science and economics.

The remainder of this paper is structured as follows: The impact of the epidemic on the trade of medical products is discussed analytically in “Section 2”; the data sources and empirical framework are illustrated in “Section 3”; the empirical results, analysis of heterogeneity, and consideration of political relations are presented in “Section 4”; robustness and endogeneity tests are elucidated in “Section 5”; finally, a summary is presented in “Section 6”.

## 2. Conceptual framework

Bilateral trade has been affected by the pandemic in the importing country, exporting country, and for other trading partners of the importing country.

### 2.1. Importing countries

On the domestic supply side of the importing country, the COVID-19 epidemic has reduced the health status of the working population, leading to a short-term shortage of labor. On the domestic demand side of the importing country, due to the unpredictability and high infectiousness of the novel coronavirus (16), the public health crisis caused by it aroused the precautionary savings motivations and rational consumption tendencies of residents. Additionally, the policy and economic uncertainty mitigated the demand for non-essential goods (17). However, in the context of the epidemic, medical products, especially personal protective products, have become immediate-need products for residents; the increasing effect of demand was significantly greater than the inhibiting effect (10), resulting in a sharp increase in public health expenditure (18). The decline in the domestic supply capacity of medical products and rise in consumer demand have caused a large imbalance between the supply and demand of importing countries, thus making countries rely on imports to meet domestic demand. Noteworthily, the influence mechanism on supply may exhibit different characteristics at different times. In the early stages of the outbreak, owing to the sudden onset of the health crisis, governments were struggling to deal with the treatment, prevention, and control of new cases, and the medical manufacturing industry did not receive sufficient policy protection or its capacity growth could not keep pace with the short-term demand growth, thus increasing its dependence on imports. However, since July 2020, the impact of the epidemic has tended to stabilize (12); therefore, the government has adopted policy measures to ensure the recovery of production for the medical manufacturing industry or promote the localized production of medical products through subsidies and other means. The inhibiting effect of the epidemic on the supply side of importing countries gradually weakened with the recovery of production capacity.

### 2.2. Exporting countries

The impact of the epidemic on both supply and demand also applies to exporting countries. The spread of the epidemic will inhibit the product supply and increase domestic demand for medical products, which will correspondingly reduce exports. However, China gradually resumed production and exports in March and April 2020 (19), and the excess capacity was released after the epidemic stabilized, effectively easing the pressure of the export reduction caused by the rising demand for domestic medical products. Therefore, the inhibiting effect of the epidemic on China's exports is predominantly derived from the government's control measures on production.

### 2.3. Other trading partners of the importing country

Anderson and van Wincoop (20) elucidated the change in multilateral trade costs, which affects the bilateral trade flows between two countries. For the other trading partners of the importing country, the pandemic exerted pressure on export reduction, increased trade costs with the importing country, and exhibited a certain trade transfer effect. Medical products originally imported by other partners may have been imported from China.

### 2.4. Political factors

Political factors should be considered when importing and exporting key medical products. As mentioned by Sutter et al. (21), the Chinese government assumed control of the production and distribution of medical products in February 2020, transferring control from the Ministry of Information Industry and Technology to the National Development and Reform Commission, the most powerful central economic planning agency. This not only improved the efficiency of domestic production and distribution of medical products but also strengthened the control over imports and exports (22, 23). Whether the Chinese government will use trade means to achieve policy objectives as before is a concern among several scholars. For instance, Verma (24), White (25), and Wong (26) reported on China's “mask diplomacy,” while Fuchs et al. (12) demonstrated that desirable political relations between countries and China helped import medical products in the early stages of the epidemic. Therefore, based on previous studies, a longer time span is considered here.

## 3. Materials and methods

### 3.1. Data

The monthly export data on medical products from China to other countries covering the period from January 2020 to September 2022 from the Chinese Customs Statistics website were used in this study. For China, using free on board (FOB) export data rather than costs-, insurance, and freight (CIF) import data can avoid the time delay caused by transportation and customs clearance, and reflect the impact of the epidemic more directly. The WTO classifies medical products into the following four categories: pharmaceuticals (including immunization products, vaccines, and medicines for human use), medical consumables (including consumables for use in hospitals and laboratories), medical equipment (including medical, surgical, and laboratory disinfectors, as well as medical and surgical instruments and equipment), and personal protective equipment (including hand sanitizers and disinfectants, masks, and protective glasses). This study classifies these into three categories—namely, critical medical products (labor-intensive products), drugs (knowledge-intensive products), and medical equipment (capital-intensive products)—to better reflect the distribution characteristics of China's export products. China's exports of medical products are dominated by labor-intensive products, followed by knowledge-intensive products, while its capital-intensive products are less competitive than those of developed countries (27).

Combined with the characteristics of the data presented on the website of China Customs, non-medical products (e.g., industrial raw materials) were excluded from the list of epidemic prevention materials released by the China General Administration of Customs, and 34 medical product categories under the HS8-digit code were retained to form the critical medical product dataset, including medical masks, disposable protective clothing, test kits, vaccines, alcohol disinfection, ventilators, along with medicines in the CODES-30 category and medical equipment under the HS9018-HS9022 classification.

Based on the literature, specifically, Hayakawa and Mukunoki (1), and Liu et al. (15), the promoting effect on the demand side of a country and inhibiting effect on the supply side are expressed by the intensity of the epidemic and strict control of the government, respectively. These two variables are endogenous to each other: The epidemic triggers strict control, while a strong lockdown policy curbs the spread of the epidemic. Therefore, this study only controls for two effects simultaneously in the importing country to consider their independent influences and only for one variable in the exporting country and other partner countries.

Data from the Oxford COVID-19 Government Response Tracker were used to measure the intensity of a country's epidemic on a monthly basis and strictness of government control, by systematically collecting the daily new infections and deaths since the outbreak of the epidemic, as well as the open policy information of the government in response to the epidemic (28). The intensity of the epidemic in the exporting countries was expressed by smoothing the new cases per million people per month. The stringency of government controls was measured using the stringency index in the dataset. The higher the stringency index, the more restrictions the government placed on domestic economic activities, such as the closure of workplaces and schools, and consequently, the more constrained the supply capacity of domestic products. The indicators above are all daily data in the dataset, which are summarized and converted into monthly data through frequency conversion.

Referring to Liu et al. (15), the construction method of epidemic indicators affecting multilateral trade costs is proposed, and the transaction value of product *m* in 2019 is considered the weight to calculate the average epidemic severity of other trading partners of the importing country. The formula is as follows:


(1) $$Covid\_tpc_{imt}=\frac{\sum_{j=1}^{N}Trade_{ijm,2019}Covid_{jt}}{\sum_{j=1}^{N}Trade_{ijm,2019}}$$

where weighted item *Trade*_*ijm*, 2019_ represents the transaction value of product *m* between importing country *i* and trading partner countries other than China in 2019. As there is no relevant classification of key medical products in 2019, the weight of such products is the same as that of drugs because the former were classified drugs before the outbreak of the epidemic. The 2019 transaction value data used for weighting were derived from the BACI-CEII database.

Finally, for measuring political connection, the annual dataset of “political distance” provided by Bailey et al. (29) has been used, which utilizes the item response theory model to estimate the political distance of the ideal point based on the voting preferences of countries at the annual UN General Assembly. The larger the value is, the greater is the corresponding political distance. This index is widely used to measure border political relations (30, 31).

### 3.2. Econometric model

After deleting the observations with excessive trade zeros and excessive missing statistical values, a dataset of 5,214 observation points covering 33 months in 158 countries was obtained. Based on the expansion of the traditional trade gravity model, we express the benchmark model as follows:


(2) $$\begin{array}{ll} Trade_{ijt}= & \beta_{0}+\beta_{1}Covid_{\text{i}t}+\beta_{2}Stringency_{it}+\beta_{3}Stringency_{jt}\\ & +\beta_{4}Covid\_ptc_{it}+\delta_{i}+\delta_{t}+\varpi_{it} \end{array}$$

where *i* represents the importing country; *t* represents the month; and *j* represents the exporting country (China). *Trade*_*ijt*_ is the explained variable, representing the value of product *m* imported from China by country *i* in month *t*. The core explanatory variables are *Covid*_*it*_ and *Stringency*_*it*_, representing the outbreak and government control situation of the importing country in month *t*, represented by the monthly number of new infections per million and Stringency_*jt*_ index of country *i*, respectively. These two variables are endogenous. An increase in the number of infected people will precipitate stricter control by the government, but strict lockdowns also control the epidemic's development. The two variables are included in the model to explore their independent impacts on the domestic demand and supply. *Stringency*_*jt*_ represents the severity of the measures adopted by the Chinese government in response to the outbreak, without simultaneously controlling for the number of new cases, as in importing countries, to avoid multicollinearity. *Covid_ptc*_*it*_ represents the average severity of COVID-19 in the importing countries, except for China. δ_*i*_ is a national fixed effect used to control for the influence of some time-invariant differences (e.g., population size, population aging degree, and geographical distance) between countries. δ_*t*_ is a time-fixed effect controlled at the monthly level to eliminate the seasonal effects and changes in the total welfare of the world economy, and ω_*it*_ is a random perturbation term.

To investigate the change in the epidemic impact over time, a monthly dummy variable was introduced into Equation (2) as follows:


(3) $$\begin{array}{l} Trade_{ijyt}\;=\;\alpha_{0}+\alpha_{1}Covid_{iyt}D^{\prime }+\alpha_{2}Stringency_{iyt}D^{\prime }\\ \;+\alpha_{3}Stringency_{jyt}D^{\prime }+\alpha_{4}Covid\_ptc_{iyt}D^{\prime }\\ \;+\delta_{i}+\delta_{y}+\varpi_{ijyt} \end{array}$$

*Trade*_*ijyt*_ represents the value of medical products imported from China by importing country *i* in month *t* of year *y*; *D'* is a dummy variable used to indicate the month; and δ_*y*_ is the year-fixed effect.

Finally, the variable for “political distance,” which measures political relationships, is introduced and a set of year fixed effects is added to eliminate the interference of the year trend:


(4) $$\begin{array}{l} Trade_{ijyt}\;=\;\beta_{0}+\beta_{1}Covid_{\text{iy}t}+\beta_{2}Stringency_{iyt}+\beta_{3}Stringency_{jyt}\\ \;+\beta_{4}Covid\_ptc_{iyt}+\beta_{5}Podis_{i(y-1)}\\ \;+\delta_{i}+\delta_{t}+\delta_{y}+\varpi_{ijyt} \end{array}$$

*Trade*_*ijyt*_ represents the value of medical products imported by country *i* from China in year *y* and month *t*, while *Podis*_*i*(*y*−1)_ represents the political distance between country *i* and China calculated by the UN voting preference in year *y*. As trade and political relations in the same year will affect each other, while political relations affect imports and exports, trade friction may also cause the deterioration of diplomatic relations; hence, political distance data are processed one period behind. This is also relevant because UN votes reflect a certain lag in the movement of political relations. δ_*y*_ represents the year-fixed effect.

Finally, all data were logarithmically processed. Standard errors were clustered at the national level using heteroscedasticity robust standard errors.

## 4. Empirical results

### 4.1. Baseline regression results

The regression results of Equation (2) are presented in Table 1; the results are reported by the category of medical products.

**Table 1:** Baseline regressions.

	**Critical medical products**		**Drugs**		**Medical equipment**	
	**M1**	**M2**	**M1**	**M2**	**M1**	**M2**
	**(1)**	**(2)**	**(3)**	**(4)**	**(5)**	**(6)**
lncovid_i	0.044^***^	0.036^***^	0.068^***^	0.057^***^	0.03^***^	0.024^**^
	(0.008)	(0.008)	(0.018)	(0.018)	(0.012)	(0.011)
lnstringency_i	0.119^***^	0.111^***^	0.265^**^	0.254^**^	0.204^***^	0.192^***^
	(0.026)	(0.026)	(0.102)	(0.1)	(0.06)	(0.058)
lnstringency_j		−1.69^**^		−6.123^***^		−2.555
		(0.673)		(2.015)		(1.658)
lncovid_ptc		0.108^***^		0.144^*^		0.146
		(0.034)		(0.075)		(0.092)
FE	^√^	√	√	√	√	√
Month dummies	√	√	√	√	√	√
Observations	5,214	5,214	5,214	5,214	5,214	5,214
R-squared	0.355	0.362	0.171	0.174	0.135	0.138

Standard errors are between parentheses. ^***^p < 0.01, ^**^p < 0.05, ^*^p < 0.1.

The results for critical medical products, drugs, and medical equipment are reported here, and epidemic variables of importing countries and other epidemic variables are added into the model in two steps, with results reported step by step for M1 and M2. Country- and month-fixed effects are controlled for all categories.

Core explanatory variables *Covid*_*it*_ and *Stringency*_*it*_ are both significantly positive for the three product categories, indicating that COVID-19 in importing countries exhibits a significant promoting effect on the import of medical products from China, confirming that COVID-19 impacts both supply and demand in importing countries, resulting in a surge in import demand.

Among the first two types of products, *Stringency*_*jt*_ of the government control in the exporting country China is significantly negative. Multilateral trade resistance item *Covid_ptc*_*it*_ is significantly positive, demonstrating the inhibiting effect of COVID-19 on exporting countries and the trade diversion effect on multilateral trade. However, these two variables do not have significant coefficients for the third category of products for two main reasons. First, most medical equipment (e.g., ventilators) are capital-intensive commodities with high technological content. Compared to developed countries in Europe and the United States, China's medical equipment is less competitive, meaning importing countries are more inclined to import from other countries. Second, medical equipment is a durable good.^3^

In conclusion, the regression results verify the tripartite channels through which the epidemic affected the trade of medical products: (1) The epidemic in importing countries generally promoted the import of medical products from China. (2) The government control caused by the epidemic in China limited its ability to export medical products. (3) The epidemic in other trading partners of China restricted its exports, resulting in a trade diversion effect and an increase in the import of key medical products and medicines from China.

### 4.2. Analysis of period heterogeneity

In this study, the epidemic is divided into two phases to explore heterogeneity—namely, the outbreak phase and plateau phase. To accurately select the cutoff point of a period, a monthly dummy variable is introduced into Equation (3) to observe the significant difference in the epidemic impact for each month. The regression results are presented in Table 2, focusing on the coefficient and joint significance of the time dummy variable.

**Table 2:** Monthly level estimation.

	**Key medical products**	**Drugs**	**Medical equipment**
	**(1)**	**(2)**	**(3)**
lncovid_i	0.036^***^	0.057^***^	0.024^**^
lnstringency_i	0.111^***^	0.254^**^	0.192^***^
lncovid_j	−2.213^***^	−6.192^*^	−0.794
lnstringency_ptc	0.108^***^	0.144^*^	0.146
Dmonth202001	−0.105	−0.014	0.355
Dmonth202002	−1.552^***^	−0.822^*^	−1.328^***^
Dmonth202003	−0.65^***^	0.228	−0.775^***^
Dmonth202004	−0.714^***^	−1.897^**^	−0.963^**^
Dmonth202005	0.264^***^	−0.073	−0.203
Dmonth202006	0.468^***^	0.513^*^	0.089
Dmonth202007	0.273^***^	0.466	−0.052
Dmonth202008	0.064	0.158	−0.158
Dmonth202009	−0.696^***^	−1.431^**^	−0.655
Dmonth202010	−0.772^***^	−1.52^**^	−0.61^**^
Dmonth202011	−0.55^***^	−0.664^*^	−0.607^***^
Dmonth202012	−0.132	0.106	−0.651^*^
Dmonth202101	−0.248^**^	−0.005	−0.688^**^
Dmonth202102	−0.645^***^	−0.162	−0.949^***^
Dmonth202103	−1.185^***^	−1.859	−0.936
Dmonth202104	−0.232^***^	0.555^***^	−0.442^**^
Dmonth202105	−0.464^***^	0.004	−0.637^***^
Dmonth202106	−0.221^***^	0.572^***^	−0.38^***^
Dmonth202107	−0.179^*^	0.835^***^	−0.358^***^
Dmonth202108	−0.266^***^	0.511^**^	−0.174^*^
Dmonth202109	−0.219^***^	0.779^***^	−0.242
Dmonth202110	−0.253^***^	0.634^***^	−0.217^*^
Dmonth202111	−0.256^***^	0.309	−0.395^**^
Dmonth202112	−0.187^**^	0.84^***^	−0.222
Dmonth202201	0.088	0.19	−0.224
Dmonth202202	−0.9^***^	−1.924^***^	−1.219^***^
Dmonth202203	−0.674^***^	−1.361^**^	−0.489
Dmonth202204	−0.19^*^	−0.239	−0.425^***^
Dmonth202205	0.064	0.207	−0.128
Dmonth202206	0.198^**^	0.766^**^	0.182
Dmonth202207	0.028	0.263^**^	−0.133
Observations	5,214	5,214	5,214
R-squared	0.357	0.172	0.136

Standard errors are between parentheses. ^***^p < 0.01, ^**^p < 0.05, ^*^p < 0.1.

There are several significant regression coefficients for key medical and pharmaceutical products, indicating that the impact of the outbreak varied widely from month to month. By comparing the sizes of the coefficients, it is not difficult to determine that, in the first few months after the outbreak of the epidemic, the coefficients changed greatly for the same significance level, stabilized for the first time in the last quarter of 2020, and then gradually decreased. Therefore, this can preliminarily indicate that the impact of the outbreak tended to stabilize during this period.

**Figure 1:**
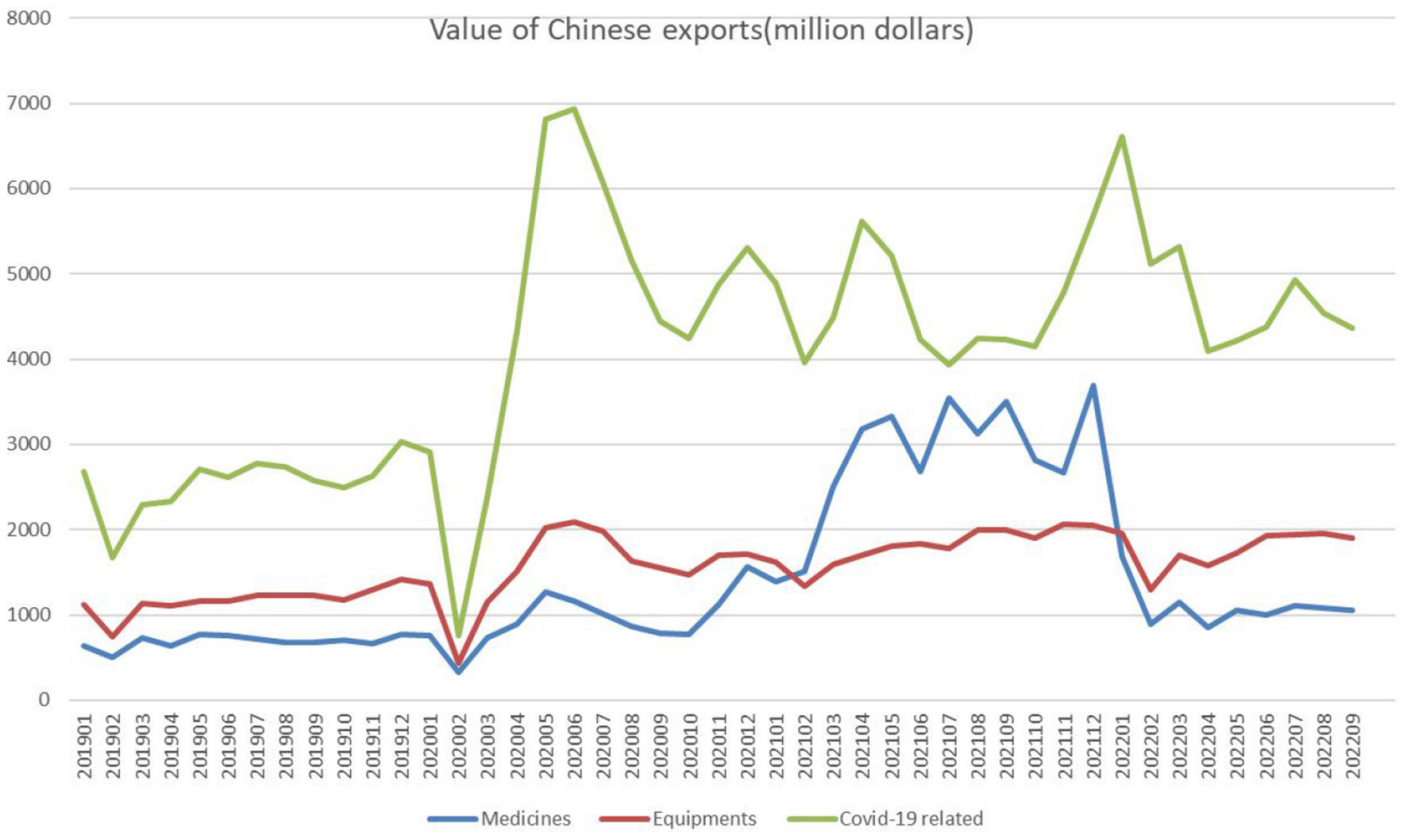
Chinese exports of medical goods. Source: Authors' computations using data from China's general administration of customs.

Additionally, Figure 1 presents China's medical product exports for each month since 2020 and the changing trend. The export value of key medical products peaked in June 2020, returned to the first trough in October, and remained fluctuating at this level in the following months. In conclusion, it is reasonable to consider October 2020 as the critical point, with the “outbreak period” before October and “plateau period” after it.

Next, observations from the outbreak period were removed, and a set of 24 months from October 2020 to September 2022 during the platform period was obtained to focus on the characteristics of the epidemic impact during the platform period. For reference, we also selected the sample set of the first 24 months including the outbreak period, and the consistent sample numbers in both cases made the regression results comparable. The regression results are reported in Table 3.

**Table 3:** Time differences.

	**Key medical products**		**Drugs**		**Medical equipment**	
	**T1**	**T2**	**T1**	**T2**	**T1**	**T2**
	**(1)**	**(2)**	**(3)**	**(4)**	**(5)**	**(6)**
lncovid_i	0.043^***^	0.038^***^	0.091^***^	0.065^**^	0.027^*^	0.027
	(0.01)	(0.009)	(0.027)	(0.026)	(0.015)	(0.016)
lnstringency_i	0.095^***^	0.102^***^	0.31^***^	0.224^*^	0.236^***^	0.093
	(0.029)	(0.038)	(0.119)	(0.123)	(0.055)	(0.066)
lnstringency_j	−2.381^***^	3.975^***^	−4.927^**^	5.635^**^	−3.822	3.168^***^
	(0.752)	(0.652)	(2.093)	(2.237)	(2.457)	(1.054)
lncovid_ptc	0.097^***^	0.116^***^	0.177^**^	0.126	0.129	0.125
	(0.036)	(0.037)	(0.07)	(0.092)	(0.113)	(0.093)
FE	√	√	√	√	√	√
Month dummies	√	√	√	√	√	√
Month 01/2020–12/2021	√		√		√	
Month 10/2020–09/2022		√		√		√
Observations	3,792	3,792	3,792	3,792	3,792	3,792
R-squared	0.406	0.084	0.207	0.12	0.163	0.039

Standard errors are between parentheses. ^***^p < 0.01, ^**^p < 0.05, ^*^p < 0.1.

In Table 3, T1 and T2 correspond to the regression results for the first and second 24 months of the epidemic (plateau period), respectively. T2 had less significant coefficients than T1, and the goodness of fit also decreased significantly. In conclusion, although the epidemic continues, its impact on imported medical products has gradually subsided during the plateau period.

Compared with the other two categories of products, critical medical products exhibited the least significant loss, and the impact of the epidemic was still significant during the platform period. On the one hand, key medical products are the most directly related to the epidemic. Owing to the requirements of epidemic prevention and control, even if the infection rate decreases, countries will not significantly reduce their stockpiles of epidemic prevention materials, such as masks, vaccines, and test kits. On the other hand, China is a major exporter of epidemic prevention materials, accounting for a large proportion of global exports. In 2020, global personal protective equipment (PPE) exports increased by 44.6%, while China's PPE exports increased by 208%,^4^ and imports over the plateau period were still predominantly from China. However, in terms of medical equipment, owing to China's weak competitiveness and the fact that such products are not used for epidemic prevention and control and are only durable goods used for treatment, the demand decreased during the plateau period; consequently, the impact of the epidemic almost completely lost its statistical significance.

The coefficients on the importing country's severity index for the last two product categories are significantly reduced in absolute and significant terms, while the effects of multilateral trade almost completely disappeared. This is because, as the epidemic entered the second stage, the domestic production capacity of each country gradually recovered, and the government implemented measures to prioritize the production of domestic medical products. To consider national security, some governments accelerated the localized production of medical products (e.g., Turkey reapplied the garment manufacturing industry to the production of PPE). Therefore, the suppressive effect of the epidemic on the domestic supply side of countries has been significantly weakened by policy protection.

Noteworthily, the coefficients on China's severity index for the three products all become positive and significant in the second stage. This indicates that, after the outbreak, China's strict domestic control measures did not inhibit the production and export of medical products. This is due to the fact that the Chinese government centralized the production and distribution rights of medical products in February 2020, and the central authorities conducted macro-control, which ensured that the production and distribution of medical products were unaffected by the lockdown measures.

In summary, the main conclusions are as follows: First, after entering the stabilization period, the impact of the epidemic in three aspects on the import of non-critical medical products evidently reduced, while the impact on the import of key medical products continues. Second, the negative effect of domestic control measures in exporting country China on exports completely disappeared during the epidemic plateau.

### 4.3. Political factors

In Equation (4), political distance was included in the model to explore the role of political relations in exporting medical products to China. The estimated results are reported by product category and period in Table 4. T1, T2, and T3 correspond to the full stage of the epidemic, first 24 months, and second 24 months of the epidemic, respectively.

**Table 4:** Political distance regressions.

	**Key medical products**			**Drugs**			**Medical equipment**		
	**T1**	**T2**	**T3**	**T1**	**T2**	**T3**	**T1**	**T2**	**T3**
	**(1)**	**(2)**	**(3)**	**(4)**	**(5)**	**(6)**	**(7)**	**(8)**	**(9)**
lncovid_i	0.038^***^	0.043^***^	0.043^***^	0.057^***^	0.092^***^	0.066^**^	0.023^**^	0.028^*^	0.024
	(0.008)	(0.009)	(0.009)	(0.018)	(0.027)	(0.026)	(0.011)	(0.014)	(0.016)
lnstringency_i	0.106^***^	0.095^***^	0.087^**^	0.253^**^	0.31^***^	0.219^*^	0.194^***^	0.236^***^	0.1
	(0.025)	(0.029)	(0.037)	(0.1)	(0.119)	(0.122)	(0.058)	(0.055)	(0.067)
lnstringency_j	−1.716^**^	−2.37^***^	3.92^***^	−6.127^***^	−4.946^**^	5.616^**^	−2.548	−3.827	3.21^***^
	(0.667)	(0.751)	(0.647)	(2.018)	(2.089)	(2.239)	(1.664)	(2.457)	(1.043)
lncovid_ptc	0.11^***^	0.097^***^	0.123^***^	0.144^*^	0.177^**^	0.129	0.145	0.129	0.124
	(0.034)	(0.036)	(0.037)	(0.075)	(0.07)	(0.093)	(0.092)	(0.113)	(0.093)
Podis	0.284^***^	0.178	0.321^***^	0.041	−0.304	0.111	−0.139	−0.078	−0.158
	(0.093)	(0.146)	(0.098)	(0.252)	(0.368)	(0.263)	(0.121)	(0.18)	(0.135)
FE	√	√	√	√	√	√	√	√	√
Month dummies	√	√	√	√	√	√	√	√	√
Year dummies	√	√	√	√	√	√	√	√	√
01/2020–09/2022	√			√			√		
01/2020–12/2021		√			√			√	
10/2020–09/2022			√			√			√
Observations	5,214	3,792	3,792	5,214	3,792	3,792	5,214	3,792	3,792
R-squared	0.359	0.406	0.089	0.172	0.208	0.12	0.137	0.163	0.039

Standard errors are between parentheses. ^***^p < 0.01, ^**^p < 0.05, ^*^p < 0.1.

In Table 4, the coefficient on the political distance variable is only significant for the key medical products, which indicates that the export of key medical products has a stronger political meaning than the other two categories.

Contrary to the findings of existing studies, the coefficient on this variable is significantly positive for critical medical products, suggesting that China's exports of key medical products tend to favor countries with distant UN voting distances throughout the pandemic, which is inconsistent with the general practice in the rest of the world during the outbreak period^5^ (32). After considering the different phases of the epidemic, the influence of political relations in the first phase was not significant, whereas in the second phase, the influence was extremely significant, and the absolute value of the coefficient significantly increased. The explanation is that, as Fuchs et al. (12) elucidated, China exported more key medical products to countries they had desirable good political relations with in the early stages of the outbreak; however, after the epidemic entered the stabilization period, China adjusted its export strategy and tried improving international diplomatic relations through trade means.

## 5. Robustness checks

### 5.1. Index measurement method with changed explanatory variables

New confirmed cases per million population were used in the model to measure the occurrence of the epidemic. However, different countries have different diagnostic capabilities for COVID-19, leading to variations in the measurement of this indicator. Therefore, this index was replaced by the number of new deaths per million in the same dataset, and Equation (2) was re-estimated. A comparison with the previous results is presented in Table 5.

**Table 5:** New death and new case regressions.

	**New cases**			**New deaths**		
	**C1**	**C2**	**C3**	**C1**	**C2**	**C3**
	**(1)**	**(2)**	**(3)**	**(4)**	**(5)**	**(6)**
lncovid_i	0.036^***^	0.057^***^	0.024^**^	0.059^***^	0.099^***^	0.027
	(0.008)	(0.018)	(0.011)	(0.011)	(0.027)	(0.017)
lnstringency_i	0.111^***^	0.254^**^	0.192^***^	0.106^***^	0.245^**^	0.192^***^
	(0.026)	(0.1)	(0.058)	(0.025)	(0.107)	(0.059)
lnstringency_j	−1.69^**^	−6.123^***^	−2.555	−0.911	−4.837^**^	−2.135
	(0.673)	(2.015)	(1.658)	(0.657)	(2.068)	(1.667)
lncovid_ptc	0.108^***^	0.144^*^	0.146	0.097^***^	0.122	0.149
	(0.034)	(0.075)	(0.092)	(0.031)	(0.081)	(0.095)
FE	√	√	√	√	√	√
Month dummies	√	√	√	√	√	√
Observations	5,214	5,214	5,214	5,214	5,214	5,214
R-squared	0.362	0.174	0.138	0.359	0.173	0.136

Standard errors are between parentheses. ^***^p < 0.01, ^**^p < 0.05, ^*^p < 0.1.

C1, C2, and C3 correspond to key medical products, drugs, and medical devices, respectively. “New deaths” correspond to the regression results after replacing the indicators. Compared with “new cases,” although there is a certain lag in the use of new deaths to measure the occurrence of the epidemic (the COVID-19 virus does not cause immediate death), the direction, magnitude, and significance of the variable coefficients have not changed dramatically. As such, the conclusions regarding the impact of the pandemic have not changed.

Additionally, the analysis of the influence of political relations herein is based on the index of “ideal point distance,” which is a modified version of the UN voting preference record by Bailey et al. (29). To avoid the influence of the indicator construction method on the results, the original data in the dataset “United Nations Voting Similarity Index” (Agreements) were used to replace previous voting distance data. The higher the index is, the closer is the political position; the key medical products are re-estimated using Equation (4). A comparison of the results is presented in Table 6.

**Table 6:** Ideal point distance and agreements.

	**Ideal point distance**			**Agreements**		
	**T1**	**T2**	**T3**	**T1**	**T2**	**T3**
	**(1)**	**(2)**	**(3)**	**(4)**	**(5)**	**(6)**
lncovid_i	0.038^***^	0.043^***^	0.043^***^	0.036^***^	0.043^***^	0.038^***^
	(0.008)	(0.009)	(0.009)	(0.008)	(0.01)	(0.009)
lnstringency_i	0.106^***^	0.095^***^	0.087^**^	0.11^***^	0.095^***^	0.101^***^
	(0.025)	(0.029)	(0.037)	(0.026)	(0.028)	(0.038)
lnstringency_j	−1.716^**^	−2.37^***^	3.92^***^	−1.742^**^	−2.522^***^	3.959^***^
	(0.667)	(0.751)	(0.647)	(0.669)	(0.748)	(0.653)
lncovid_ptc	0.11^***^	0.097^***^	0.123^***^	0.11^***^	0.101^***^	0.116^***^
	(0.034)	(0.036)	(0.037)	(0.034)	(0.036)	(0.037)
Podis	0.284^***^	0.178	0.321^***^	−0.43^**^	−1.398	−0.116
	(0.093)	(0.146)	(0.098)	(0.204)	(1.06)	(0.235)
FE	√	√	√	√	√	√
Month dummies	√	√	√	√	√	√
Year dummies	√	√	√	√	√	√
01/2020–09/2022	√			√		
01/2020–12/2021		√			√	
10/2020–09/2022			√			√
Observations	5,214	3,792	3,792	5,214	3,792	3,792
R-squared	0.359	0.406	0.089	0.358	0.407	0.084

Standard errors are between parentheses. ^***^p < 0.01, ^**^p < 0.05.

T1, T2, and T3 correspond to the time spans and “Agreements” reports the regression results with replacement indicators and results for the full phase, first 24 months, and second 24 months of the epidemic, respectively. In the full-stage regression, a significant negative coefficient was still present, and the sign of the coefficient did not change during the phased regression, indicating that, over the entire epidemic period, China exhibited a tendency to export to countries with relatively different political positions in the United Nations. This further supports the conclusion that China improved its international relations through the export of key medical products.

### 5.2. Discussion on endogeneity

Additonally, a strong endogenous relationship possibly exists between the COVID-19 epidemic in importing countries and the imports of critical medical products. On the one hand, the spread of the epidemic has caused an increase in the import demand for medical products. On the other hand, as an endless supply of masks, protective suits, vaccines, and other epidemic prevention materials was shipped to importing countries, the epidemic prevention and control capacity of importing countries did improve, thus affecting the epidemic situation. This two-way causal relationship cannot be ruled out. Therefore, this study solves this problem by constructing tool variables for a two-stage least squares (2SLS) regression.

Liu et al. (33) used the genetic distance between other countries and China in 1500 AD to construct instrumental variables to solve the endogeneity problem when studying the impact of the epidemic on world imports and exports from January to June 2020. On the one hand, genetic inheritance is relatively stable, and the ancient genetic relationship between countries and China is likely to continue today, thus affecting the physiological genetic similarity between people from other countries and China today. The higher the similarity is, the more likely people are to be infected with COVID-19, which satisfies the instrumental variables' correlation condition. However, the historical genetic distance has nearly no impact on current trade. Liu et al. (33) removed all data from China and the reference of the genetic distance in the sample to further meet the homogeneity requirements of the instrumental variables.

As this study only examines imports from China and cannot eliminate observations, it further explores the channel—geographical distance—through which historical genetic distance may affect current trade. Giuliano et al. (34) demonstrate that, at least in the case of trade flows, genetic distance represents the same geographical factors that lead to genetic differences among different populations. Therefore, in this study, the geographical distance factor is separated from the individual fixed effect in Equation (3), and the core explanatory variables are combined to form a cross-multiplying term, which is introduced into the model for regression. The results are presented in Table 7.

**Table 7:** Geographical distance.

	**Key medical products**	**Drugs**	**Medical equipment**
	**(1)**	**(2)**	**(3)**
lncovid_i	0.039^***^	0.048^***^	0.018
	(0.009)	(0.017)	(0.013)
lnstringency_i	0.117^***^	0.237^***^	0.182^***^
	(0.029)	(0.086)	(0.058)
lnstringency_j	−1.41^*^	−6.867^**^	−2.995^*^
	(0.0723)	(2.677)	(1.692)
lncovid_ptc	0.106^***^	0.148^*^	0.147
	(0.034)	(0.075)	(0.092)
Dist^*^lncovid_i	−0.009	0.024	0.015
	(2.964)	(0.032)	(0.013)
FE	√	√	√
Month dummies	√	√	√
Observations	5,214	5,214	5,214
R-squared	0.355	0.172	0.137

Standard errors are between parentheses. ^***^p < 0.01, ^**^p < 0.05, ^*^p < 0.1.

The results of the three products are reported in this table respectively. It is not difficult to determine that the cross-term of geographical distance and epidemic in the three products is not significant, and the coefficient is extremely small. In conclusion, geographical distance does not affect the import of medical products from China. Therefore, the homogeneity of genetic distance as an instrumental variable is further proved.

In summary, this study provides a new interaction term based on the genetic distance between the populations of different countries and China in 1500 AD and the logarithm of the core explanatory variable “per million newly confirmed cases,” which is used as an instrumental variable for the 2SLS regression. The regression results for key medical products are reported in Table 8.

**Table 8:** 2SLS regression.

	**First stage**	**Second stage**
	**(1)**	**(2)**
lncovid_i^*^gendis	18.665^***^	
	(0.236)	
Lncovid_i		0.035^***^
		(0.009)
lnstringency_i	0.002	0.11^***^
	(0.039)	(0.024)
lnstringency_j	−1.015	−2.232^*^
	(1.714)	(1.239)
lncovid_ptc	0.393^***^	0.109^***^
	(0.037)	(0.031)
FE	√	√
Month dummies	√	√
KP rk LM statistic	1151.498^***^	
KP rk Wald F statistic	6256.854 <16.38>	
Hansen J statistic	0.000	
Observations	5,181	5,181
R-squared		0.357

Standard errors are between parentheses. ^***^p < 0.01, ^*^p < 0.1.

The two columns in the table are the results for the first and second phases of the 2SLS. In the first stage, the instrumental variables were significantly positively correlated with the outbreak status, indicating that the closer is the genetic distance to China, the stronger is the outbreak degree, proving the rationality of the instrumental variables. In the results of the second stage, the significance of the coefficient, the size, and the direction of the value exhibit no significant changes compared with the results of the baseline regression, which indicates the reliability of the estimated results. Additionally, the statistical values of LM, Wald F, and HJ exhibit no under-recognition, weak recognition, or over-recognition of the instrumental variables, thereby confirming their effectiveness.

In summary, after considering the measurement error, index construction method, and two-way causality, the results still exhibit no significant changes, which proves the estimated results' robustness.

## 6. Conclusions

This study empirically examined the situation of 159 countries importing medical products from China from January 2020 to September 2022. Horizontally, the impact of the epidemic on the trade of medical products in importing countries, exporting countries, and other trading partners has been considered, which fully covers all aspects of the impact of the epidemic on trade, rather than only some aspects, as in previous studies; further, the product classification has been improved according to the technical level of the products, considering labor-intensive key medical products, knowledge-intensive drugs, and capital-intensive medical equipment. This classification method combines the characteristics of China's medical product exports, rather than the general classification according to international standards, thus making the analysis more targeted. In terms of time, October 2020 divided the epidemic into the outbreak period and platform period. This is the first study to discuss the epidemic in different periods, which makes it possible to observe changes in the impact of the epidemic, thus making it possible to observe all aspects of the impact of the epidemic on the imports and exports of medical products to obtain microscopic and objective conclusions.

The findings can be summarized as follows: First, the aggravation of the epidemic situation in the importing country will reduce domestic production and increase the demand for medical products, which will increase the import demand and promote the import of medical products from China. Second, the strongest response is in relation to key medical products (e.g., masks, protective clothing, test kits), followed by medical products (e.g., vaccines, basic drugs), and finally medical equipment (e.g., ventilators), which is also positively related to the proportion of the various products in China's exports. Third, if the epidemic situation of other trading partners in the importing country became serious, trade cost increases and trade diversion effect occurred, which promoted China's exports. Fourth, after the outbreak, the gradual recovery of the national production capacity and gradual maturity of policies reduced the intensity of the import demand. The overall impact of the epidemic on the import of medical products has weakened; however, this weakening is not evident for key medical products. Fifth, China's domestic epidemic has a limited inhibitory effect on the export of medical products, and strong macro-control has ensured the production and export of key medical products. Finally, over the entire epidemic period, China did not export more key medical products to countries with similar political positions, as numerous scholars had predicted, but tended to export to countries with minimal political distance. The Chinese government used the exports of key medical products to improve foreign relations.

These findings provide a realistic basis for global public health governance in the post-epidemic era. Although the epidemic has been ongoing for 3years and epidemic prevention and control have become normal, the importance of medical product trade—as the ballast stone for all mankind to fight against virus invasions—cannot be ignored. Owing to the strong response of the medical product trade to the epidemic, countries should adopt stricter macro-control measures to ensure the stability of the cross-border supply chain of medical products, especially key medical products, to ensure that they can respond more calmly to epidemic impacts. At home, appropriate policies and measures should be taken to ensure that the production of medical products is not affected. Countries should try to reduce the restrictions on the resumption of work and production of the medical industry in the state of pandemic prevention. In terms of international trade, goverments should pay attention to the smooth entry of medical products under the epidemic situation, and establish a “green channel” when appropriate to ensure that the import and export of medical products are not hindered. Additionally, the trade of medical products has acquired political significance during the epidemic. Countries should, thus, maintain an open attitude and strengthen cooperation with other countries. Therefore, to effectively battle the pandemic, which is a war for all of mankind, global cooperation is a necessity.

There are still some deficiencies in this article: First, Because there are a large number of trade zeros in the samples, this paper deletes a large number of samples, resulting in a significant decline in the estimation accuracy. PPML estimation method can be used to solve this problem in future research. Second, the data of many variables cannot be included in the model because they are not updated in time, and can only be absorbed by fixed effects. The following research can separate the variables such as public health costs and hospital beds from the fixed effect to ensure the consistency of the estimated results. Finally, After the epidemic entered the platform period, many countries released the control of the epidemic and abandoned the official statistics of new infections and deaths, leading to the loss of reference significance of some data used in the article. This is not discussed in this paper, which can be taken into account in future research.

## Data availability statement

The raw data supporting the conclusions of this article will be made available by the authors, without undue reservation.

## Author contributions

Material preparation, data collection, and analysis were performed by YP and AX. The first draft of the manuscript was written by YP. All authors contributed to the study conception and design, commented on previous versions of the manuscript, and read and approved the final manuscript.

## Footnotes

^1^ http://www.scio.gov.cn/zfbps/32832/Document/1681809/1681809.htm

^2^ https://www.wto.org/english/tratop_e/covid19_e/trade_related_goods_measure_e.htm

^3^ https://www.wto.org/english/tratop_e/covid19_e/med_goods_2019_21_e.pdf

^4^ https://www.wto.org/english/tratop_e/covid19_e/med_goods_2019_21_e.pdf

^5^Hayakawa and Imai's (32) study revealed that the exporters of medical products were negatively affected by the outbreak in its early stages, but this effect was weakened among countries with close political ties.
